# Novel 3-Methyl-2-alkylthio Benzothiazolyl-Based Ionic Liquids: Synthesis, Characterization, and Antibiotic Activity

**DOI:** 10.3390/molecules23082011

**Published:** 2018-08-12

**Authors:** Teng He Zhang, Hao Xi He, Jun Liang Du, Zhi Jian He, Shun Yao

**Affiliations:** 1 School of Chemical Engineering, Sichuan University, Chengdu 610065, China; zthrainbow@foxmail.com; 2 Mianyang Blood Center, Mianyang 621000, China; pharmposter2012@163.com; 3 College of Chemistry and Chemical Engineering, Mianyang Normal University, Mianyang 621000, China; 15928065204@139.com

**Keywords:** 3-methyl-2-alkylthio benzothiazolyl-based ionic liquids, solubility, thermal stability, antibiotic activity

## Abstract

Three series of novel 3-methyl-2-alkylthio benzothiazolyl ionic liquids (ILs) were synthesized for the first time. After structural identification, their melting point, solubility, and thermostability together with antibiotic activity were determined successively. As a result, 3-methyl-2-alkylthio benzothiazolyl *p*-toluene sulfonate was found to have the highest antibacterial activity among the three series of ILs. Meanwhile, it has a good solubility in water as well. On the basis of comprehensive comparison with similar compounds, the effect of cations and anions of these benzothiazolium ILs on typical physical properties together with antibiotic performance was explored and discussed, which is very beneficial to take the greatest advantage of their structural designability for various purposes. Furthermore, the experiment data preliminarily discovered the relationships of the structure-properties/activities of the above three kinds ILs to a certain extent, which can provide useful references for future research and for the potential application of these new ILs as surfactant antiseptics or agricultural chemicals.

## 1. Introduction

Benzothiazole derivatives are an important kind of heterocycles which are popularly applied as a variety of pharmaceutical agents and bioactive natural products. They have received more and more attention because of their various pharmacological activities such as their antimicrobial [1], anticancer [2], anti-inflammatory [3], analgesic [4], antitubercular [5], and antidepressant activities [6], etc. Moreover, some of them have efficient catalytic activity and are used in chemical reactions and so forth [7]. With a common thermally stable electron-withdrawing nucleus, benzothiazole derivatives are a class of molecules displaying a variety of chemical and biological functions.

At present, ionic liquids (ILs) are exhibiting a series of unique properties, such as a low melting point, non-flammability, good solvation, high thermal stability and so on [8]. Meanwhile, ILs possess an ideal designability for their structures, which are promoting people to create more new ILs based on various nuclei and substituent groups, and the innovation of their structures always results in new bioactivity. For instance, the biocidal behaviors of larger organic cations are commonly used because of their inhibition of bacterial, fungal or insect growth [9,10,11,12]. Among them, 1-[(1R,2S,5R)-(−)-menthoxymethyl]-3-alkylimidazolium chlorides demonstrated an extremely high activity against microbes with a wide spectrum of activity and alkoxymethyl(2-hydroxyethyl)dimethylammonium acesulfamates have been shown to be potential insect feeding deterrents. Especially, the series of ILs with imidazolium nucleus have been widely investigated by a great number of researchers and related work has made a lot of progress [13]. The mechanism behind their antimicrobial action is also considered similar to that of cationic surfactants. Moreover, a good correlation of the alkyl chain length in the cations of 1,3-dialkylimidazolium ILs with their cytotoxicity in two different cell cultures and the marine bacteria *V. fischeri* has been found [14]. Moreover, some researchers reported and employed a series of important classes of ionic liquids for their toxicity towards both prokaryotic and eukaryotic microorganisms [15]. By comparison, it was found the imidazolium ion shows higher toxicity towards freshwater algae than the pyridinium ion [16,17]. However, no research work has been published these bioactivities of the ionic liquids with benzothiazolium cation together with related sulfhydryl substituents, and a little study only focused on their synthesis, physical properties and catalytic application (e.g., [18]). In recent years, some reviews have revealed that many effective antimicrobial agents have a heterocyclic segment within their structure [19]. Generally, the preceding mercaptobenzothiazole (MBT) derivatives have good biological activities [20]. However, most of them have a very poor solubility in water, so their application is limited. Whether the assembled ILs can generate different antimicrobial activity or other new biological activities needs to be explored. For the above considerations, it was planned in this study to introduce the substituted benzothiazole group into ionic liquids for the synthesis of 3-methyl-2-alkylthiobenzothiazolyl ILs with three kinds of anions. According to online search with SciFinder Scholar (Chemical Abstract Service), 13 compounds (**A1**–**A5**, **B1**–**B5** and **C1**–**C3**) have not been reported. 

## 2. Results and Discussion

### 2.1. Synthesis Method and Conditions

**Scheme 1:**
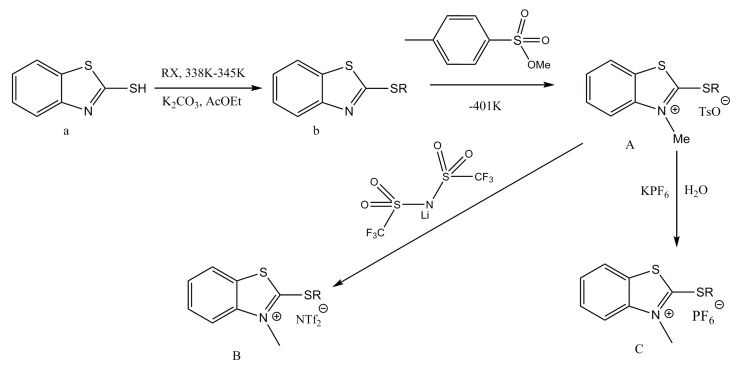
The synthetic routes of 3-methyl-2-alkylthio benzothiazolyl-based ILs (R = CH_3_-, C_2_H_5_-, n-C_4_H_9_-, n-C_6_H_13_-, n-C_8_H_17_-, benzyl groups).

In a previous study, the antimicrobial activity of 23 newly synthesized pyridinium ILs was tested against a panel of bacteria and fungi [21]. The results proved that all tested ILs were effective antibacterial and antifungal agents and that the inhibition zone of these ILs with PF_6_^−^ is 13.4–20.9 mm (MIC = 3.9–125 µg/mL). More recently, the antimicrobial activity of 24 ILs paired with different anions (hydrophilic anions: Cl, Br, SCN, DCA, and BF_4_; hydrophobic anion: NTf_2_; amino acid anions: Gly, Ala, Ser, Pro, and Asn) have been investigated and the results clearly demonstrated that introduction of the hydrophobic anion of bis((trifluoromethyl)sulfonyl)amide (NTf_2_^−^) and elongation of the cations substitutions could result in higher bioactivity (EC_50_ = 0.31–3.86 Mm) [8]. Therefore, the combination of benzothiazolium cations with three anions of OTs^−^, PF_6_^−^, and NTf_2_^−^ was finally selected in this study. The synthetic route of 13 kinds of ILs is shown in Scheme 1, and these ionic liquids were obtained successfully through the three-step approach as follows:

(1) 2-alkylthio benzothiazole (compound **b**) was firstly obtained via a nucleophilic substitution reaction between MBT and alkyl bromide;

(2) 3-methyl-2-alkylthio benzothiazolyltosilate (compound **A**) was obtained by the reaction between 2-alkylthiobenzothiazole and the 4-methyl benzene sulfonic acid methyl ester;

(3) 3-methyl-2-alkylthiobenzothiazolyl salts with NTf_2_^−^ and PF_6_^−^ (compounds **B** and **C**) were synthesized lastly by anion exchange reaction. 

There are two reaction sites in MBT, which include sulfydryl and amino. Li et al. [22] found that lower temperature were more beneficial for the sulfydryl substituent, and higher temperature were more advantageous to the amino substitution reaction for MBT when other conditions were the same. In summary, the temperature of the reaction plays an important role in the thiol alkylation reaction of MBT. In our experiment, the synthesis reaction of compound **b** was conducted for nearly 10 h at 343 K or 18 h at 333 K. If the temperature was lowered than 313 K, the obvious reaction would not have been observed within 24 h. Through comparison of various reaction conditions, the range of 338–343 K was finally selected as the appropriate temperature range for the synthesis of **b1**–**b5**, and the yield of **b** reached 90.35–96.48%. After the reaction, excessive inorganic salts were removed by filtration. Then the filtrate was washed with a saturated Na_2_CO_3_ aqueous solution and water successively. The residual solvent and water were removed under a vacuum to obtain a crude product, which was further purified with silica gel column chromatography using 5 to 10% (*v*/*v*) EtOAc in petroleum ether (boiling range: 30–60 °C) as an eluent to afford pure 2-methylthio benzothiazole. 

In the synthesis process of compound **A**, if the temperature was below 353 K, the reaction time was doubled. When it was higher than 408 K, the byproducts of the reaction would increase and then the purity of **A** would decrease. The optimal temperature of the synthesis reaction of **A** was determined to be 403 K after repeated experiments. As a result, a 0.02 mol product **b** reacted with equimolar *p*-methyltoluene sulfonate at 403 K for 6 h, and the viscous fluid product was obtained when the system was cooled to room temperature. The viscous fluid product was washed with deionized water four times at 393 K. Then the aqueous solution was merged and centrifuged, and the upper light yellow water layer was isolated after demulsification. Finally, the product was dehydrated under vacuum and further dried with magnesium sulfate to give product **A**. As shown in Table 1, the yield of **A1**–**A5** was in the range of 97–68%. It was found that the yield of **A1**–**A5** reduced with an increase of the carbon chains of thiol alkyl at the same reaction temperature, which resulted from the increase of the steric hindrance of the carbon chain of thiolalkyl.

In the last step, compound **A** (0.01 mol) was dissolved in 20 mL of water and a transparent homogeneous solution was obtained. A total of 25 mL of LiNTf_2_ aqueous solution (0.0105 mol) was added dropwise to the solution of compound **A** and the mixture was stirred for 12 h. After the reaction, a light yellow solid product at the bottom of the flask was separated out from the reaction mixture, and then it was washed with deionized water for four times and recrystallized in ethyl acetate, successively. The product was dried and obtained as compound **B**.

### 2.2. Spectral Data and Analysis

The data of IR, ^1^H NMR, ^13^C NMR, ^19^F NMR, and ESI-MS of all the synthesized ILs are shown in the Supplementary Materials, which can be used to identify their structures and related substituted groups through comparison with the spectral information from the previous study of benzothiazolium ILs [23]. As an example, the four signals of δ 8.15 (1H), 7.93 (1H), 7.72–7.68 (1H), 7.59–7.56 (1H) in ^1^H NMR (600 MHz, CDCl_3_) of compound **A1** ((3-Me-2-S-C1-MBT)(OTs)) can be assigned to the four protons in the benzene ring in the benzothiazolium nucleus, and the two peaks at δ7.65 (2H) and 7.06 (2H) can be assigned to the protons of the AA′BB′ system in the benzene ring of the anion. The signals at δ4.17 (s, 3H), 3.06 (s, 3H), and 2.30 (s, 3H) are attributable to the three -CH_3_ groups connected with N, S, and O atoms, respectively. The corresponding carbon signals can also prove the existence of above nucleus and groups. In its IR spectra, an absorbance at 3092.6 and 3023.6 cm^−1^ can be assigned to the stretching vibration of aromatic hydrogens in the benzene rings of the cation and anion, which also cause the signals below 1000 cm^−1^ because of bending vibrations. The stretching and bending vibrations of the various methyl groups result in several peaks in the range of 3000–2900 cm^−1^ and 1500–1400 cm^−1^, respectively; the stretching vibration region of the benzenesulfonyl group in the anion exists in the range of 1260–1000 cm^−1^, and the wavenumber of the asymmetric stretching vibrations is higher than that of the symmetric one. The absorbance in the range of 1600–1400 cm^−1^ belongs to the skeleton vibration of the benzene rings and the C=N signal usually appears around 1600 cm^−1^. Furthermore, the Finnigan TSQ Quantum Ultra LC/MS/MS system (Thermo Fisher, San Jose, CA, USA) in a dual ion pattern and full-scan mode (100–500 *m*/*z*) was operated to determine the cations and anions (see Figure S1 in Supplementary Materials) under the following conditions: N_2_dryinggas with a flow rate of 700 L·h^−1^ and 400 °C, a 3.0 kV capillary voltage, and a 100 °C capillary temperature. The MS bars of the three various anions appear at *m*/*z* 171 (TsO^−^), 280 (NTf_2_^−^), and 145 (PF_6_^−^), as calculated, successively. Moreover, it was found that the mass to charge ratio of the cations increases with the growth of their alkyl length. As a conclusion, MS can be used to discriminate them rapidly.

### 2.3. Physical State and Melting Points of Synthesized ILs

As known to all, as a kind of organic molten salts displaying ionic-covalent crystalline structures, ILs can remain in a liquid status over a very wide temperature range (e.g., 233.15–423.15 K) and has a low melting point (≤423.15 K). Generally, a working definition is that an ionic liquid is a salt with a melting temperature below the boiling point of water. Seddon et al. [24] also hold the opinion that there is nothing sacred about a temperature of 100 °C and that it is merely a convenient and arbitrary marker. The melting point is one of the important properties of ILs, which depends on the different compositions of cations and anions. The total lattice energy of the ionic compounds is higher and their melting point is higher. Various combinations of cation and anion will have significant effects on the melting point of the ILs on the basis of their volume, structural symmetry, planarity, H-bond, induction effect, and so on [25]. As shown in Table 2, it can be observed that the ILs of **A1**–**A5** have lower melting points than the other two series of ILs when their cations are the same. The melting points of **B1**–**B4** decrease with the increasing length of the alkyl chain of the cation, which have the same anion of (NTf_2_^−^). However, if the length of the alkyl chain of the cation was much longer, the melting point would rise up (such as **B5**, 78.9–79.5 °C). This U-type trend is the same as that stated in previous reports on the melting points of immidazolium ILs with different alkyl side chains [26]. The results show that the symmetry of the cation of the ILs will become lower with the increasing length of the alkyl chain of the cation. If the length of the alkyl chain of the cation is further increased, it will result in the increase of the intermolecular van der Waals force and the aggregation among the lipophilic groups in the structures of different ILs. When the cation is the same, PF_6_^−^ has the smallest volume among the three kinds of anions, which leads to a higher crystal stacking density and a higher lattice energy. The order of the anion volume is as follows: OTs^−^ > NTf_2_^−^ > PF_6_^−^, so their melting points are significantly different. 

### 2.4. Solubility Investigation

The solubility behavior of ILs in common solvents always play key roles in their usage. Based on previous study [16], many hydrophilic acesulfamate-based ILs were antimicrobially active. In this section, the solubility values of all obtained ILs were evaluated in eight common solvents at 298.0 ± 0.5 K, which was investigated using Al-mohammed′s method [27]. The results were defined as miscible (marked with +), partially miscible (marked with ±), or immiscible (marked with −), respectively [28]. Generally, ionic compounds have an ideal solubility in polar solvents on the basis of the “like dissolves like” theory, and there is a certain relationship between their dissolution behavior and the dielectric constant (ε) of the related solvents. According to the results of the solubility test shown in Table 3, it was found that some ILs can be dissolved in the solvents with moderate or low polarity, except in *n*-hexane; and the individual cations and anions can be tunable to produce ILs with the desired solubility. For instance, the type of anion plays a strong impact on the solubility of ILs in water [29], those ILs with NTf_2_^−^ or PF_6_^−^ are immiscible with water, which can hardly provide H-bond interaction with hydrone and always appear as hydrophobic anions by researchers in the previous study on immidazolium ILs. All of the ILs with OTs^−^ are immiscible with ethyl acetate, but the ILs combined with NTf_2_^–^ are miscible with ethyl acetate. All the studied ILs are moderately miscible or miscible with acetone, which has an unsaturated and polarized carbon–oxygen bond and can provide more intermolecular interaction than protic solvents. When their polarity and dielectric constant further declines, the solvents only have the selective dissolving capacity for a part of the tested ILs. Finally, it can be concluded that the dispersion force between these ILs and alkane is weaker than that between alkane molecules. Therefore, they are not miscible with *n*-hexane.

### 2.5. Thermal Stability

Considering the potential conditions in their application, the thermal stability of these ILs was evaluated in terms of the decomposition temperature, which is determined with a TGA/SDTA851 thermogravimetric analyzer (Metrohm, Switzerland) with a heating rate of 10 °C/min from 30 to 450 °C under a nitrogen atmosphere. Their thermal stability is influenced by the interactions between the carbon atom and the heteroatom or the heteroatom and the H-bond [30]. The structures, together with properties of the cation and anion, are closely related with the decomposition temperature of the ILs [31]. As a result, the TGA thermograms of **A1**–**A5** IL (see Figure S2 in the Supplementary Materials) show two common weight loss regions, successively. The first region is within the temperature range of 50–145 °C, and the weight loss of the ILs in this range is 7–17 wt %. This weight loss is ascribed to the evaporation of residual water, and compounds **A1**–**A5** have a strong hygroscopicity at room temperature, which is in accordance with the fact that they have a higher solubility in water. The second stage is from 145 to 350 °C, the onset decomposition temperature of **A1**–**A5** is 145 °C and they were decomposed completely around 350 °C. The degradation of **A1**–**A5** with a 20–80% total weight loss within the above range of decomposition temperatures is due to the breakage of the nuclear structure of these ILs. Figure S2c–f display the thermogravimetric analysis results of the ILs and the two series of **B** and **C** (e.g., **B1**–**B5** containing NTf_2_^−^). Generally, the coordination, affinity, and hydrophilicity of the anions are closely related with the thermostability of ILs. As a result, it can be concluded that the order of thermal stability of anions is NTf_2_^−^ > OTs^−^ > PF_6_^−^. ILs containing NTf_2_^−^ are the most promising candidates for higher temperature application, while the thermal stability of ILs with OTs^−^ is weaker than those with NTf_2_^−^ because of the electron-donating effect of methyl on the benzene ring reduces the force constant of the C-S bond of the sulfonic acid group. On the other hand, the relationship curve of thermal stability of the cations and the total carbon number of the side chain on the cation zigzags. The cation of 3-methyl-2-alkylthio benzothiazolyl has a worse thermostability than the immidazolium cation for its more unsaturated structure, and the latter usually begins to degrade at temperatures above 350 °C. Of course, the maximum applicable temperature of these ionic liquids depends on the duration of the applications and tolerance to changes in quantity and quality.

### 2.6. Antibiotic Activity

Finally, the antibiotic effect of ILs was also investigated by testing their activity against some disease-causing crop bacteria with different antimicrobial resistance profiles. The related data of this study were the average values of the three parallel experiments unless there is special explanation. The results of the inhibitory zone diameter (mm) are summarized in Table 4. Through careful observation and comparison, it can be found that the two series of **A** and **B** all have obvious antibiotic effects compared against the blank control groups. Furthermore, the inhibitory zone diameters (mm) of the ILs of **A** are obviously wider than those of the ILs of **B**, so the antibiotic function of the ILs of **A1**–**A5** is more ideal than the series of **B**. Compared with the other study on the antimicrobial activity of benzoazole ionic liquids (an 8 mm inhibition zone against *Gram-positive B. subtillis* for [C_6_mim][TBT] and a 9 mm inhibition zone against *P. aeruginosa* for both [C_4_mim][TBO] and [C_2_mim][TBI]) [32], the **A** series has more ideal antibiotic activity.

Then the minimum inhibitory concentrations (MIC) of the ILs were determined, which were used to compare both the antibacterial activities of the **A** and **B** series of ILs against representative standard strains of Gram-positive and Gram-negative bacteria by the tube double dilution method [33]. For comparison, a common aminoglycoside antibiotic (neomycin) and an important natural antibacterial compound (emodin) were selected as positive controls, which are the representatives of water-soluble and lipid-soluble agents with conjugated unsaturated structures, respectively. Three parallel experiments were carried out in each group and the relevant data were processed to obtain Table 5. The various combinations of different cations and anions could significantly affect the biological activities of the target ILs [34]. Considering that the structure of ILs is similar to surfactants and cationic surfactants possessing interfacial properties, these ILs can also injure the membrane of common cellular structures by attacking the lipid structure of the lipo-polysaccharide membrane of microorganisms with the alkyl groups on their cations [35]. On the one hand, the effective concentration of hydrophobic ionic liquids in water is not enough to produce a significant impact on the cell membrane, so this could result in lower activity. On the other hand, the ionic liquids depend on hydrophobic groups to combine with the phospholipid bimolecular layer of the microbial cell membrane to change its structure and permeability. The hydrogen on the sulfydryl of **A1**–**A5**/**B1**–**B5** ILs was replaced by the alkyl groups with various lengths, which could result in considerable difference in the antibacterial functions of the two series of ILs. For example, the antibacterial action of **A1**–**A5** increased successively against *Staphylococcus aureus*, *Xanthomon* *asaxonopodispv. citri*, and the *Ginger bacterial wilt strains* with the increase of the total number of carbons in the side chain (see Table 4). All the previous studies have also proved that each ionic liquid has the most appropriate alkyl chain length for a specific microorganism. It cannot be simply concluded that the longer the alkyl chain is, the higher their activity is. When the cation is the same, the anion in the ILs will show a significant influence on their biological activity (see Table 5). The MIC values of typical cationic surface-active fungicides and antimicrobials usually range from 8 to 500 μg·mL^−1^, and their inhibitory effects are realized by attacking the cell membrane and making it lose permeability. Here, the MIC values of related ILs against *Staphylococcus aureus* can prove the antibacterial activity of OTs^−^ is stronger than that of NTf_2_^−^. Compounds **A1**–**A5** have higher biological activities against *Staphylococcus aureus* than against *Escherichia coli*. It can be noticed that neomycin shows the lowest MIC value for the two bacteria, which is usually used to treat related microorganisms in crops. However, the bacteria can develop resistance to it very easily after exposure to neomycin, which is gradually being restricted from being used in the field. More importantly, its stability is very unsatisfactory; e.g., solutions of neomycin are significantly unstable to heat, which will change its color above 30 °C. Thus, it is very unsuitable for outdoor anti-microorganism use. On the contrary, related benzothiazolium ILs are relatively stable, and the antibacterial activity of **A3**–**A5** is obviously higher than that of emodin and **A1**/**A2**, which should be ascribed to the contribution of the alkyl side chain their cations. 

## 3. Materials and Methods

### 3.1. Reagents and Methods

The compounds 2-mercaptobenzothiazole (MBT, purity: 98%), methyl iodide (purity: 99%), bromine ethane (purity: 98%), 1-bromobutane (purity: 99%), 1-bromohexane (99%), 1-bromooctane (purity: 98%), benzylbromide (purity: 99%), 4-methyl-benzenesulfonicacimethylester (purity: 99%), bis (trifluoromethane) sulfonamidelithium salt (purity: 99%), and sodium hexafluorophosphate (purity: 98%) were purchased from the Borhett chemical technology company (Chengdu, China). All the solvent and reagents were used without further purification.

Column chromatography was implemented using HG/T2354-92 silica gel (Haiyang Chemical Co. Ltd., Qingdao, China) with various specified eluents. Thin layer chromatography was carried out on 0.15–0.2 mm layer silica coated HSGF 254 plates (Haiyang Chemical Co. Ltd., Qingdao, China). The IR spectra were recorded on JASCO, Bomem MB, and Shimadzu-8400S FTTR spectrophotometers. The Finnigan TSQ Quantum Ultra LC/MS/MS system (Thermo Fisher, San Jose, USA) in a dual ion pattern and full-scan mode (100–500 *m/z*) was operated to determine the cations and anions under the following conditions: N_2_ drying gas with a flow rate of 700 L·h^−1^ and a temperature of 400 °C, a 3.0 kV capillary voltage, and a 100 °C capillary temperature.^1^H NMR and ^13^C NMR spectra were recorded on a Bruker 600 spectrometer in CDCl_3_. The thermal stability of ILs was evaluated in terms of their decomposition temperature by using a TGA/SDTA851 thermogravimetric analyzer (Metrohm, Switzerland). 

### 3.2. Synthesis of 3-Methyl-2-alkylthio Benzothiazolyl-Based Ionic Liquids

MBT (5.017 g, 0.03 mol), bromomethane (2.848 g, 0.03 mol) and anhydrous potassium carbonate (4.140 g, 0.03 mol) were mixed and stirred in ethyl acetate at 65 °C. The reaction was monitored by thin layer chromatography (TLC). After the completion of the reaction, the inorganic salts that had precipitated from the reaction mixture were removed by filtration, the filtrate was washed with aqueous saturated Na_2_CO_3_ and water successively, and then it was dried by MgSO_4._ Finally, MgSO_4_ was removed by filtration; the filtrate was concentrated by reduced pressure distillation and then purified by silica gel column chromatography using 5 to 10% EtOAc in petroleum ether (*v*/*v*) as an eluent to obtain pure 2-methylthio benzothiazole (**b1**). The above general method was also used for the synthesis of different compounds of **b2**–**b5**. 

Product **b1** (3.625 g, 0.02 mol) and 4-methyl benzene sulfonic acid methyl ester (3.724 g, 0.02 mol) were reacted at 130 °C for 6 h, and then a very sticky fluid was obtained after the system was cooled to room temperature. It was washed four times with water at 20 °C and then the aqueous solution was merged and centrifuged to eliminate emulsification. The upper layer was collected and concentrated by reduced pressure distillation and then the product **A1** was obtained. The preparation method of **A2**–**A5** was analogous to that of **A1**.

**A1** (3.665 g, 0.01 mol) was dissolved completely in water and a LiNTf_2_ (3.014 g, 0.0105 mol) aqueous solution was added dropwise to above solution. After stirring for 1 h, oil droplets began to appear at the bottom of the flask and a light yellow solid was separated out from the reaction mixture after stirring for 12 h. After filtration, the filter cake was washed with water for four times and dried under a vacuum. The crude product was recrystallized in ethyl acetate and then the product **B1** was obtained. The preparation method of **B2**–**B5** was analogous to that of **B1**. The preparation method of **C1**–**C2** was analogous to that of **C1**. **A1** (3.665 g, 0.01 mol) was dissolved completely in water and asodium hexafluorophosphate (1.7635 g, 0.0105 mol) aqueous solution was added dropwise into the solution of **A1**. After stirring for 13 h, a yellow solid was finally separated from the reaction mixture. After filtration, the filter cake was washed by water four times and dried under vacuum. The crude product was recrystallized in acetone and then the product **C1** was obtained. The preparation method of **C2**–**C3** was analogous to that of **C1**.

### 3.3. Antibacterial Activity

The antibacterial activity of ILs (**A1**–**A5** and **B1**–**B5**) against 6 kinds of bacteria was evaluated by the modified Oxford cup method [36] and the inhibition zone diameters were measured. Beef extract peptone agar media was used for the bacterial culture. A total of 1 mL of an 18-hour-old culture was added to 60 mL of the medium, and 20 mL of this culture was shaken and placed into sterile Petri dishes. After the solidification of the agar medium, the sterile stainless steel cylinders (6 × 10 mm) were placed on the surface of the seeded agar and filled with 100 mL of the tested samples at 1 mg·mL^−1^. The plates were incubated at 37 °C for 24 h.

Commercial antibiotics—neomycin and emodin—were used as positive controls with the same range of concentrations. Distilled water was used as a negative control to dissolve all the tested ILs in a concentration range of 0.0165–0.250 mg·mL^−1^. An overnight culture grown in the beef extract peptone broth was diluted to approximately 10^8^ CFUmL^−1^ in sterile water. The diluted bacterial suspension was inoculated onto agar plates containing serial two-fold dilutions of neomycin and tested ILs at a final concentration ranging from 250 to 15.6 μg·mL^−1^ [37]. The MIC was defined as the lowest concentration of antibiotic that was able to prevent visible organism growth after incubation for 16 to 18 h at 37 °C.

## 4. Conclusions

In summary, a total of 13 novel 3-methyl-2-alkylthiobenzothiazolyl ionic liquids (ILs) in three series were synthesized and first reported. Their melting point, solubility, thermostability, and typical biological activities were determined. The results of this bioactivity research indicated that compounds **A3**–**A5** had an interesting antibacterial activity with the lowest MIC value of 16.7μg·mL^−1^ against *Staphylococcus aureus*, which was better than natural antibacterial compounds. Moreover, ILs **A3**–**A5** are easily soluble in water and more stable than common aminoglycoside antibiotics, which make them have favorable application foreground.

## Figures and Tables

**Table 1:** The structures, yields, and other information of the prepared ILs.

Entry	ILs Structure	ILs abbr.	M.W. (HR-MS)	Yield/%	Reaction Time/h
**A1**	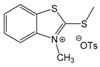	[3-Me-2-S-C1-MBT][OTs]	367.5064	97	6
**A2**	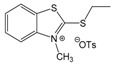	[3-Me-2-S-C2-MBT][OTs]	381.5328	95	6
**A3**	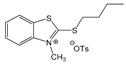	[3-Me-2-S-C4-MBT][OTs]	409.5856	85	6
**A4**	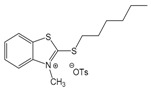	[3-Me-2-S-C6-MBT][OTs]	437.6389	72	6.5
**A5**	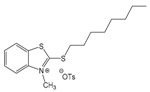	[3-Me-2-S-C8-MBT][OTs]	465.6920	68	7
**B1**	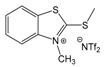	[3-Me-2-S-C1-MBT][NTf_2_]	476.4586	95	12
**B2**	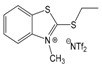	[3-Me-2-S-C2-MBT][NTf_2_]	490.4852	93	12
**B3**	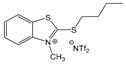	[3-Me-2-S-C4-MBT][NTf_2_]	518.5380	95	13
**B4**	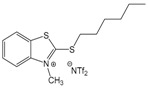	[3-Me-2-S-C6-MBT][NTf_2_]	546.5915	99	13
**B5**	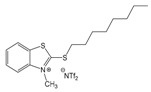	[3-Me-2-S-C8-MBT][NTf_2_]	574.6445	96	13
**C1**	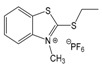	[3-Me-2-S-C2-MBT][PF_6_]	355.3031	90	11
**C2**	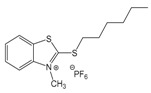	[3-Me-2-S-C6-MBT][PF_6_]	411.4093	94	11
**C3**	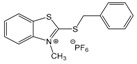	[3-Me-2-S-Bn-MBT][PF_6_]	417.3726	93	11

**Table 2:** The state and melting points of the synthesized ILs.

Entry	State under Room Temperature	Entry	Melting Point/°C	Entry	Melting Point/°C
**A1**	Brown viscous liquid	**B1**	78.9–79.9	**C1**	146.4–147.3
**A2**	Brown viscous liquid	**B2**	58.1–58.8	**C2**	206.6–207.0
**A3**	Brown viscous liquid	**B3**	55.1–56.0	**C3**	171.3–171.9
**A4**	Brown viscous liquid	**B4**	49.9–50.1		
**A5**	Brown semi-solid	**B5**	78.9–79.5		

**Table 3:** The solubility of the 13 ionic liquids in various common solvents.

No.	Water	Alcohol	Acetone	Tetrahydrofuran	Ethyl Acetate	Chloroform	Toluene	*n*-Hexane
ε	80.1	25.8	20.7	7.58	7.3	5.1	2.4	1.9
**A1**	+	+	±	-	-	-	-	-
**A2**	+	±	±	-	-	+	-	-
**A3**	+	±	±	+	-	+	-	-
**A4**	+	+	+	−	-	+	−	−
**A5**	+	+	±	+	−	+	−	−
**B1**	−	±	+	+	+	+	−	−
**B2**	−	+	+	+	+	+	±	−
**B3**	−	+	+	+	+	+	−	−
**B4**	−	±	+	+	+	+	±	−
**B5**	−	-	+	+	+	+	+	−
**C1**	−	−	+	−	−	−	−	−
**C2**	−	−	+	+	+	+	±	−
**C3**	−	−	+	−	−	−	−	−

Note: Al-mohammed’s method [27] was employed for the determination of solubility. For liquid samples, (+) means miscible: a drop of the compound dissolved in a few drops (1–5) of solvent; (±) means moderately miscible: a drop of the compound dissolved in 10 drops (<2 mL) of solvent; (−) means immiscible: a drop of the compound did not dissolve in 1–2 mL of solvent. For solid samples, (+) means miscible: 50 mg of the compound dissolved in a few drops (1–5) of solvent; (±) means moderately miscible: 50 mg of the compound dissolved in 10 drops (2 mL) of solvent; (−) means immiscible: 50 mg of the compound undissolved in 1–2 mL of solvent. Measurement temperature: 298.0 ± 0.5 K.

**Table 4:** The inhibitory zone diameter s (mm) of the ILs in the **A** and **B** series.

**Sample**	***Staphylococcus Aureus***		***Escherichia coli***		***Chinese Cabbage Soft Rot Disease***	
	**Mean**	**SD**	**Mean**	**SD**	**Mean**	**SD**
**A1**	0.0	0.0	6.0	0.0	0.0	0.0
**A2**	14.0	2.0	15.0	1.0	6.7	1.2
**A3**	19.3	1.2	21.0	2.6	16.7	3.1
**A4**	24.0	3.6	19.7	1.5	16.7	0.6
**A5**	23.0	2.6	13.3	7.0	15.7	0.6
**B1**	6.7	1.2	6.0	0.0	10.7	1.2
**B2**	10.3	4.5	10.7	4.2	11.3	2.3
**B3**	9.3	3.1	10.0	2.0	8.3	4.0
**B4**	6.7	1.2	8.7	3.1	11.3	2.3
**B5**	9.3	1.2	7.3	2.3	6.7	1.2
5% Methanol (c)	0.0	0.0	0.0	0.0	0.0	0.0
10% Acetone (d)	1.0	2.4	3.0	0.0	0.0	0.0
**Sample**	***Rice Bacterial Blight***		***Xanthomonas Axonopodispv. Citri***		***Ginger Bacterial Wilt***	
	**Mean**	**SD**	**Mean**	**SD**	**Mean**	**SD**
**A1**	6.0	0.0	0.0	0.0	2.0	1.2
**A2**	6.0	0.0	11.7	1.5	6.0	0.0
**A3**	6.0	0.0	14.0	2.6	12.0	1.0
**A4**	12.3	5.5	16.3	1.2	14.0	0.0
**A5**	17.7	3.2	16.7	3.1	16.3	0.6
**B1**	6.0	0.0	6.0	0.0	6.0	0.0
**B2**	7.3	2.3	8.0	3.5	7.3	2.3
**B3**	6.0	0.0	8.0	3.5	7.3	2.3
**B4**	7.3	2.3	9.3	3.1	10.0	3.5
**B5**	6.0	0.0	6.0	0.0	6.0	0.0
5% Methanol (c)	2.0	4.2	0.0	0.0	1.0	0.0
10% Acetone (d)	3.0	4.2	1.0	0.0	2.0	0.0

Note: The concentration of **A1**–**A5** and **B1**–**B5** was 1 mg/mL. Methanol and acetone were used as blank controls. The activity of **C1**–**C3** against some disease-causing crop bacteria was not investigated due to their poor solubility.

**Table 5:** The minimal inhibitory concentration (MIC/μg·mL^−1^) of the tested ILs in the **A** and **B** series.

Samples	*Escherichia coli*		*Staphylococcus Aureus*	
	Mean	SD	Mean	SD
**A1**	>250	0.0	125	5.3
**A2**	> 250	0.0	62.5	3.1
**A3**	>250	0.0	15.6	2.3
**A4**	>250	0.0	15.6	2.3
**A5**	>250	0.0	15.6	2.3
**B1**	250	0.0	>250	0.0
**B2**	250	0.0	>250	0.0
**B3**	250	0.0	>250	0.0
**B4**	250	0.0	250	0.0
**B5**	250	0.0	250	0.0
Neomycin	3.1	1.3	2.9	1.1
Emodin	16	2.0	20.0	3.5

Note: The MIC was determined by the concentration of a sample with 100% inhibitory, and neomycin and emodin were used as positive controls. The standard stock solution of the bacterial strain was equivalent to 1 × 10^8^ colony-forming units per milliliter. The activity of **C1**–**C3** was not investigated because of their poor solubility.

## Supplementary Materials

The following are available online at http://www.mdpi.com/1420-3049/23/8/2011/s1, Figure S1 and Figure S2.
