# Supplementary material for: Novel 3-Methyl-2-alkylthio Benzothiazolyl-Based Ionic Liquids: Synthesis, Characterization, and Antibiotic Activity
Source: Molecules. 2018 Aug 12;23(8):2011. doi: 10.3390/molecules23082011 (PMC6222442; doi:10.3390/molecules23082011)
Supplement: Supplementary file 1 [file molecules-23-02011-s001.zip › molecules-339627-SI.pdf]

Novel 3-methyl-2-alkylthio benzothiazolyl-based ionic liquids: synthesis, property and biological activity

Tenghe Zhang [1], Haoxi He[2], Junliang Du[3], Zhijian He[3,*] and Shun Yao[1,*]

[1] School of Chemical Engineering, Sichuan University, Chengdu, 610065, P.R. China; zthrainbow@foxmail.com (T.H.Z); cusack@scu.edu.cn (S.Y)

[2] Mianyang Blood Center, Mianyang, 621000, China; pharmposter2012@163.com (H.X.H)

[3] College of Chemistry and Chemical Engineering, Mianyang Normal University, Mianyang, 621000, China; 15928065204@139.com (J.L.D); youngsun9802@163.com (Z.J.H)

* Correspondence: cusack@scu.edu.cn(S.Y), youngsun9802@163.com (Z.J.H)

† T.H.Z and H.X.H: These authors contributed equally to this work.

Table of contents

1. Characterization of ILs--2

2. ESI-MS spectrum of 13 ILs--9

3. Thermograms of 13 ILs---10

Characterization of ILs

*(1) Compound **A1**: [3-Me-2-S-C1-MBT][OTs]*

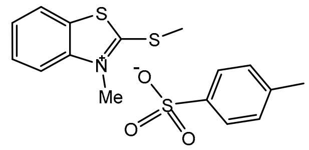

Brown viscous liquid, $C_{16}H_{17}NO_3S_3$, *M.W.*: 367.51. In ESI mass spectra, (EI^+) *m*/z calculated for $[C_9H_{10}NS_2]^+$: 196.03, found as 195.87; (EI^-) *m*/z calculated for $[C_7H_7O_3S]^-$: 171.01, found as 170.81.
IR (KBr, cm^{-1}): 3092.6, 3023.6, 2976.5, 2926.4, 1677.3, 1593.8, 1492.3, 1467.3, 1444.9, 1392.1, 1210.3, 1187.2, 1119.7, 1032.0, 1006.8, 964.2, 820.0, 759.4, 711.9, 681.7, 566.0, 428.2.
^1H NMR (600 MHz, $CDCl_3$) δ 8.15 (d, J = 8.1 Hz, 1H), 7.93 (d, J = 8.5 Hz, 1H), 7.72~7.68 (m, 1H), 7.65 (d, J = 8.1 Hz, 2H), 7.59~7.56 (m, 1H), 7.06 (d, J = 7.9 Hz, 2H), 4.17 (s, 3H), 3.06 (s, 3H), 2.30 (s, 3H).
^{13}C NMR (150 MHz, $CDCl_3$) δ 142.4, 139.3, 129.6, 128.6, 128.5, 127.4, 126.4, 125.9, 123.8, 123.2, 122.6, 115.4, 110.4, 36.9, 21.3, 18.7.

*(2) Compound **A2**: [N-Me-S-C2-MBT][OTs]*

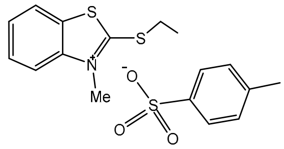

Brown viscous liquid, $C_{17}H_{19}NO_3S_3$, *M.W.*: 381.53. ESI mass spectra, (EI^+) *m*/z calculated for $[C_{10}H_{12}NS_2]^+$: 210.04, found as 209.88; (EI^-) *m*/z calculated for $[C_7H_7O_3S]^-$: 171.01, found as 170.81.
IR (KBr, cm^{-1}): 3092, 2892, 2920, 1680, 1638, 1496, 1465, 1448, 1395, 1209, 1191, 1132, 1043, 1013, 818, 763, 699, 568.
^1H NMR (400 MHz, $CDCl_3$): δ 8.13 (d, J = 8.1 Hz, 1H), 7.90 (d, J = 8.4 Hz, 1H), 7.72~7.53 (m, 3H), 7.54~7.45 (m, 1H), 7.03 (d, J = 8.0 Hz, 2H), 4.07 (d, J = 15.6 Hz, 3H), 3.68~3.27 (m, 2H), 2.27 (s, 3H), 1.88~1.21 (m, 3H).
^{13}C NMR (150 MHz, $CDCl_3$): δ 142.7, 139.1, 129.7, 128.8, 128.2, 127.2, 126.6, 125.7, 124.0, 123.0, 122.9, 115.1, 110.8, 36.7, 28.0, 21.8, 15.6.

*(3) Compound **A3**: [N-Me-S-C4-MBT][OTs]*

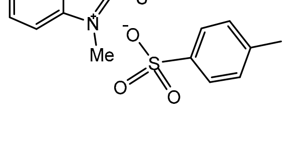

Brown viscous liquid, $C_{19}H_{23}NO_3S_3$, *M.W.*: 409.59. In ESI mass spectra, (EI^+) *m*/z calculated for $[C_{12}H_{16}S_2N_1]^+$: 238.07, found as 237.93; (EI^-) *m*/z calculated for $[C_7H_7O_3S]^-$: 171.01, found as 170.79.

IR (KBr, cm^{-1}): 3091.8, 3023.8, 2960.9, 929.7, 2871.7, 2227.1, 1674.8, 1592.8, 1493.5, 1469.0, 1447.9, 1394.1, 1325.6, 1218.7, 1186.8, 1122.0, 1032.0, 1005.8, 816.2, 757.0, 711.92, 681.2, 565.8, 498.0, 429.6.

^1H NMR (600 MHz, $CDCl_3$): δ 8.10 (dt, J = 19.5, 9.7 Hz, 1H), 7.91 (dt, J = 15.3, 7.6 Hz, 1H), 7.65 (s, 1H), 7.63 (dd, J = 12.4, 4.3 Hz,2H), 7.52~7.45 (m, 1H), 7.03 (dd, J = 13.8, 7.9 Hz, 2H), 4.10 ~4.03 (m, 3H), 3.41 (dt, J = 21.2, 7.3 Hz, 2H), 2.26 (d, J = 15.3 Hz, 3H), 1.87~1.71 (m, 2H), 1.52~1.34 (m, 2H), 0.97~0.89 (m, 3H).

^{13}C NMR (150 MHz, $CDCl_3$): δ 142.5, 139.7, 129.5, 128.6, 128.3, 127.4, 126.4, 126.0, 123.7, 123.3, 122.5, 115.6, 110.5, 37.0, 36.7, 29.7, 21.8, 21.3, 13.5.

*(4) Compound **A4**: [N-Me-S-C6-MBT][OTs]*

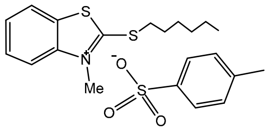

Brown viscous liquid, $C_{21}H_{27}NO_3S_3$, *M.W.*: 437.64. In ESI mass spectra, (EI^+) *m*/z calculated for $[C_{14}H_{20}NS_2]^+$: 266.10, found as 265.94; (EI^-) *m*/z calculated for $[C_7H_7O_3S]^-$: 171.01, found as 170.80.

IR (KBr, cm^{-1}): 3094.4, 3029.9, 2956.6, 2928.6, 2867.3, 1675.8, 1598.7, 1447.9, 1395.5, 1207.1, 1188.6, 1121.7, 1032.1, 1004.6, 816.6, 755.3, 681.7, 565.0, 498.1, 426.3.

^1H NMR (600 MHz, $CDCl_3$): δ 8.03 (d, J = 8.1 Hz, 1H), 7.90 (d, J = 8.5 Hz, 1H), 7.68 (dd, J = 14.5, 6.1 Hz, 1H), 7.63 (t, J = 8.1 Hz, 2H), 7.45 (t, J = 6.6 Hz, 1H), 7.08 (dd, J = 13.8, 8.1 Hz, 2H), 4.15 (s, 3H), 3.51~3.46 (m, 2H), 2.32 (s, 3H), 1.88 (dt, J = 15.1, 7.4 Hz, 2H), 1.47 (dt, J = 14.5, 7.3 Hz, 2H), 1.34~1.28 (m, 4H), 0.90 (t, J = 6.6 Hz, 3H).

^{13}C NMR (150 MHz, $CDCl_3$): δ 141.0 , 140.0 , 129.7 , 128.9 , 128.1 , 127.6, 126.4, 126.1 , 123.4 , 123.2 , 122.6, 115.5, 110.4 , 37.1, 37.0 , 31.1, 28.3, 27.8, 22.4, 21.4, 14.0.

*(5) Compound **A5**: [N-Me-S-C8-MBT][OTs]*

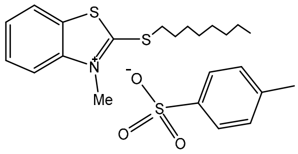

Brown viscous liquid, $C_{23}H_{31}NO_3S_3$, *M.W.*: 465.69. In ESI mass spectra, (EI^+) *m*/z calculated for $[C_{16}H_{24}NS_2]^+$: 294.14, found as 293.97; (EI^-) *m*/z calculated for $[C_7H_7O_3S]^-$: 171.01, found as 170.81.

IR (KBr, cm^{-1}): 3091.6, 3027.1, 2927.4, 2862.6, 1679.1, 1645.9, 1448.0, 1395.4, 1207.5, 1187.7, 1120.9, 1032.4, 1005.5, 817.2, 756.6, 681.9, 565.4, 506.5, 425.9.

^1H NMR (600 MHz, $CDCl_3$): δ 8.02 (d, J = 8.1 Hz, 1H), 7.90 (d, J = 8.5 Hz , 1H), 7.71~7.68 (m, 1H),

7.65 (d, J = 8.2 Hz, 2H), 7.57 (dd, J = 11.4, 4.1 Hz, 1H), 7.11 (d, J = 8.0 Hz, 2H), 4.15 (d, J = 4.5 Hz, 3H), 3.51~3.46 (m, 2H), 2.32 (s, 3H), 1.88 (dt, J = 15.1, 7.4 Hz, 2H), 1.49~1.42 (m, 2H), 1.28 (ddd, J = 29.0, 13.7, 7.9 Hz, 8H), 0.92~0.88 (m, 3H).

^{13}C NMR (150 MHz, $CDCl_3$): δ 141.2, 139.6, 137.7, 129.8, 129.0, 127.6, 126.4, 126.1, 123.2, 122.6, 122.6, 115.5, 110.4 , 37.1, 37.0 , 31.7, 29.0, 28.9, 28.7, 27.8, 22.6, 21.4, 14.1 .

*(6) Compound **B1**: [N-Me-S-C1-MBT][NTf$_2$]*

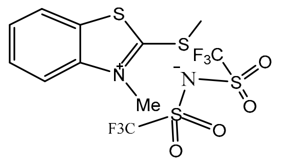

Yellow solid, m.p.78.9~79.9°C. $C_{11}F_6H_{10}N_2O_4S_4$, *M.W.*: 476.46. In ESI mass spectra, (EI^+) *m*/z calculated for [C9H10NS2]$^+$: 196.03, found as 195.89; (EI^-) *m*/z calculated for [$C_2F_6NO_4S_2$]$^-$: 279.94, found as 279.66.

IR (KBr, cm^{-1}): 3091, 3017, 2932, 1673, 1592, 1493, 1467, 1352, 1196, 1138, 1051, 791, 761, 612, 534.

^{1}H NMR (400 MHz, $CDCl_3$): δ 8.05 (d, J = 8.1 Hz, 1H), 7.88 (d, J = 8.5 Hz, 1H), 7.81 (m, 1H), 7.68 (t, J = 7.3 Hz, 1H), 4.14 (s, 3H), 3.09 (s, 3H).

^{13}C NMR (101 MHz, $CDCl_3$): δ 180.93, 142.35, 130.06, 128.06, 123.32, 121.37, 118.17, 115.24, 36.59, 18.33.

^{19}F NMR (376 MHz, $CDCl_3$): δ -78.79.

*(7) Compound **B2**: [N-Me-S-C2-MBT][NTf$_2$]*

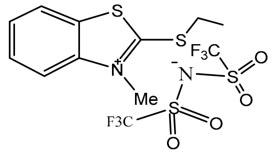

Yellow solid, m.p.58.1~58.8°C. $C_{12}F_6H_{12}N_2O_4S_4$, *M.W.*: 490.49. In ESI mass spectra, (EI^+) *m*/z calculated for [$C_{10}H_{12}NS_2$]$^+$: 210.04, found as 209.90; (EI^-) *m*/z calculated for [$C_2F_6NO_4S_2$]$^-$: 279.94, found as 279.67.

IR (KBr, cm^{-1}): 3100, 3012, 2985, 2945, 1630, 1592, 1491, 1466, 1354, 1196, 1139, 1051, 791, 761, 739, 686, 569.

^{1}H NMR (400 MHz, $CDCl_3$): δ 8.05 (d, J = 8.1 Hz, 1H), 7.88 (d, J = 8.3 Hz, 1H), 7.80 (t, J = 7.8 Hz, 1H), 7.68 (t, J = 7.6 Hz, 1H), 4.14 (s, 3H), 3.58 (q, J = 7.4 Hz, 2H), 1.69~1.56 (m, 3H).

^{13}C NMR (101 MHz, $CDCl_3$): δ 179.55, 141.98, 130.00, 127.99, 123.33, 121.38, 118.19, 115.31, 36.66, 31.55, 18.32, 13.00.

^{19}F NMR (376 MHz, $CDCl_3$): δ -78.80.

*(8) Compound **B3**: [N-Me-S-C4-MBT][NTf₂]*

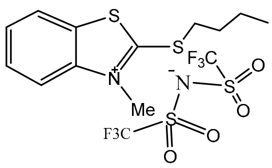

Yellow solid, m.p. 55.1~56.0°C. $C_{14}F_6H_{16}N_2O_4S_4$, *M.W.*: 518.14. In ESI mass spectra, (EI^+) *m*/z calculated for $[C_{12}H_{16}NS_2]^+$: 238.07, found as 237.91; (EI^-) *m*/z calculated for $[C_2F_6NO_4S_2]^-$: 279.94, found as 279.66.

IR (KBr, cm^{-1}): 3104, 2969, 2881, 1634, 1493, 1467, 1353, 1203, 1139, 1052, 791, 756, 740, 612, 569.

^{1}H NMR (400 MHz, $CDCl_3$): δ 8.05 (d, J = 8.1 Hz, 1H), 7.88 (d, J = 8.4 Hz, 1H), 7.79 (t, J = 7.8 Hz, 1H), 7.68 (t, J = 7.7 Hz, 1H), 4.15 (s, 3H), 3.54 (dd, J = 14.0, 6.7 Hz, 2H), 1.98 (m, 2H), 1.58 (m, 2H), 1.02 (t, J = 7.3 Hz, 3H).

^{13}C NMR (101 MHz, $CDCl_3$): δ 179.69, 142.01, 129.99, 128.00, 123.33, 121.39, 118.20, 115.30, 36.85, 36.66, 29.65, 21.80, 13.30.

^{19}F NMR (376 MHz, $CDCl_3$): δ -78.80.

*(9) Compound **B4**: [N-Me-S-C6-MBT][NTf₂]*

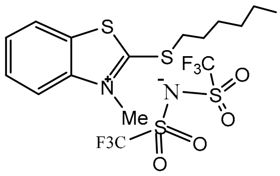

Yellow solid, m.p. 49.9~50.1°C. $C_{16}F_6H_{20}N_2O_4S_4$, *M.W.*: 546.59. In ESI mass spectra, (EI^+) *m*/z calculated for $[C_{14}H_{20}NS_2]^+$: 266.10, found as 265.95; (EI^-) *m*/z calculated for $[C_2F_6NO_4S_2]^-$: 279.94, found as 279.68.

IR (KBr, cm^{-1}): 3101, 3017, 2935, 2875, 1632, 1592, 1492, 1467, 1351, 1196, 1136, 1053, 791, 766, 617, 569.

^{1}H NMR (400 MHz, $CDCl_3$) δ: 8.05 (d, J = 8.1 Hz, 1H), 7.89 (d, J = 8.5 Hz, 1H), 7.77 (t, J = 7.9 Hz, 1H), 7.66 (t, J = 7.7 Hz, 1H), 4.12 (s, 3H), 3.51 (t, J = 7.3 Hz, 2H), 1.98 (m, 2H), 1.57~1.51 (m, 2H), 1.38~1.34 (m, 4H), 0.91 (t, J = 6.8 Hz, 3H).

^{13}C NMR (101 MHz, $CDCl_3$) δ: 179.68, 142.02, 130.00, 128.00, 127.97, 123.32, 121.39, 118.20, 115.32, 37.18, 36.68, 30.97, 28.26, 27.72, 22.34, 13.89.

^{19}F NMR (376 MHz, $CDCl_3$) δ: -78.79.

*(10) Compound **B5**: [N-Me-S-C8-MBT][NTf₂]*

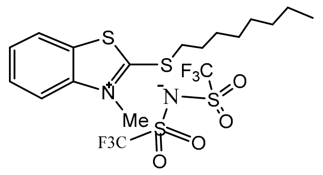

Yellow solid, m.p.78.9~79.5ᵒC. $C_{18}F_6H_{24}N_2O_4S_4$, *M.W.*: 574.64. In ESI mass spectra, (EI^+) *m*/z calculated for $[C_{16}H_{24}NS_2]^+$: 294.14, found as 293.95; (EI^-) *m*/z calculated for $[C_2F_6NO_4S_2]^-$: 279.94, found as 279.66.

IR (KBr, cm^{-1}): 3088.5, 3016.7, 2932.7, 2858.7, 1591.3, 1493.3, 14677.3, 1397.7, 1352.6, 1276.0, 1169.3, 1137.9, 1051.0, 965.9, 862.4, 793.6, 760.9, 739.5, 716.8, 705.1, 611.9, 569.6, 534.5, 514.1, 426.1.

^{1}H NMR (400 MHz, $CDCl_3$): δ 8.06 (d, J = 8.0 Hz, 1H), 7.86 (d, J = 8.5 Hz, 1H), 7.79 (t, J = 8.0 Hz, 1H), 7.68 (t, J = 7.7 Hz, 1H), 4.14 (s, 3H), 3.52 (t, J = 7.2 Hz, 2H), 1.99 (m, 2H), 1.55 (m, 2H), 1.31~1.35 (m, 6H), 0.90 (t, J = 6.5 Hz, 3H).

^{13}C NMR (101 MHz, $CDCl_3$) δ: 179.65, 142.04, 130.01, 128.02, 127.95, 123.30, 121.34, 118.18, 115.30, 37.19, 36.67, 30.93, 28.25, 27.71, 22.35, 21.41, 13.87.

^{19}F NMR (376 MHz, $CDCl_3$): δ -78.81.

*(11) Compound **C1**: [N-Me-S-C2-MBT][PF₆]*

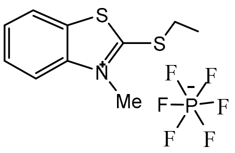

Yellow solid, m.p. 146.4~147.3ᵒC. $C_{10}F_6H_{12}NPS_2$, *M.W.*: 355.30. In ESI mass spectra, (EI^+) *m*/z calculated for $[C_{10}H_{12}NS_2]^+$: 210.04, found as 209.89; (EI^-) *m*/z calculated for $[PF_6]^-$: 144.96, found as 144.76.

IR (KBr, cm^{-1}): 3101, 3021, 2982, 2937, 1638, 1593, 1496, 1466, 1397, 1143, 838, 773, 558.

^{1}H NMR (600 MHz, $CDCl_3$): δ 8.09 (d, J = 8.2 Hz, 1H), 7.90 (d, J = 4.4 Hz, 1H), 7.82 (d, J = 4.3 Hz, 1H), 7.70 (d, J = 4.9 Hz, 1H), 4.19~4.14 (m, 3H), 3.48~3.42 (m, 2H), 1.59~1.51 (m, 3H).

^{13}C NMR (150 MHz, $CDCl_3$): δ 130.2, 130.1, 128.1, 123.5, 123.3, 115.4, 115.1, 46.8, 31.5, 13.1.

^{19}F NMR (376 MHz, $CDCl_3$) : δ -71.10.

*(12) Compound **C2**: [N-Me-S-C6-MBT][PF₆]*

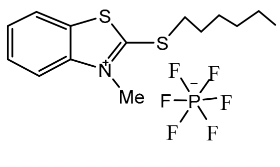

Yellow solid, m.p. 206.6~207.0ᵒC. $C_{14}F_6H_{20}NPS_2$, *M.W.*: 411.41. In ESI mass spectra, (EI^+) *m*/z

calculated for $[C_{14}H_{20}NS_2]^+$: 266.10, found as 265.95; (EI^-) *m*/z calculated for $[PF_6]^-$: 144.96, found as 144.77.

IR (KBr, cm^{-1}): 3100, 3022, 2965, 2932, 2878, 2861, 1624, 1590, 1494, 1467, 1398, 1140, 836, 760, 557.

^{1}H NMR (600 MHz, $CDCl_3$): δ 8.09 (d, J = 8.2 Hz, 1H), 7.90 (d, J = 4.4 Hz, 1H), 7.82 (d, J = 4.3 Hz, 1H), 7.70 (d, J = 4.9 Hz, 1H), 4.19~4.14 (m, 3H), 3.48~3.42 (m, 2H), 1.59~1.51 (m, 3H).

^{13}C NMR (150 MHz, $CDCl_3$): δ 130.2, 130.1, 128.1, 123.5, 123.3, 115.4, 115.1, 46.8, 31.5, 13.1.

^{19}F NMR (376 MHz, $CDCl_3$): δ -71.20.

*(13) Compound **C3**: [N-Me-S-Bn-MBT][PF$_6$]*

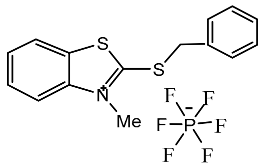

Yellow solid, m.p. 171.3~171.9°C. $C_{17}F_6H_{14}N_2O_4S_4$, *M.W.*: 417.37. In ESI mass spectra, (EI^+) *m*/z calculated for $[C_{15}H_{14}NS_2]^+$: 272.06, found as 271.90; (EI^-) *m*/z calculated for $[PF_6]^-$: 144.96, found as 144.76.

IR (KBr, cm^{-1}): 3097, 3015, 2930, 1627, 1603, 1589, 1498, 1465, 1419, 1132, 837, 767, 724, 700, 558.

^{1}H NMR (600 MHz, $CDCl_3$): δ 8.11 (d, J = 8.3 Hz, 1H), 8.02 (d, J = 8.0 Hz, 1H), 7.89 (d, J = 8.4 Hz, 1H), 7.80~7.75 (m, 1H), 7.58 (d, J = 6.6 Hz, 2H), 7.52 (d, J = 8.0 Hz, 1H), 7.07 (d, J = 8.0 Hz, 2H), 4.78 (s, 2H), 4.20 (s, 3H).

^{13}C NMR (150 MHz, DMSO): δ 141.0, 133.7, 130.2, 128.5(×2), 128.3, 127.9 (×2), 126.9, 123.3, 122.3, 115.8, 115.5, 40.8, 32.7.

^{19}F NMR (376 MHz, $CDCl_3$): δ -71.10.

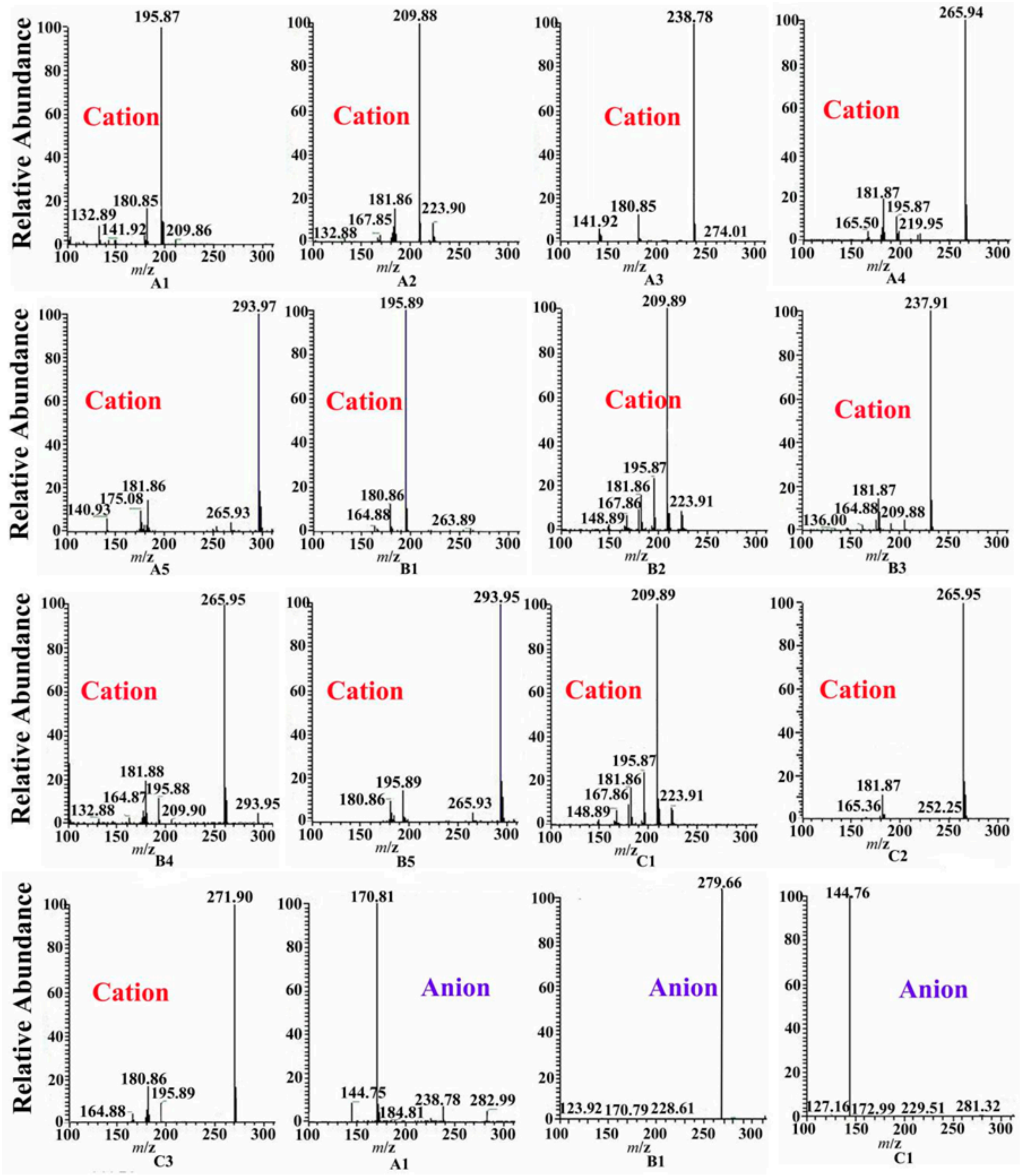

Figure S1. ESI-MS spectrum of 13 ILs.

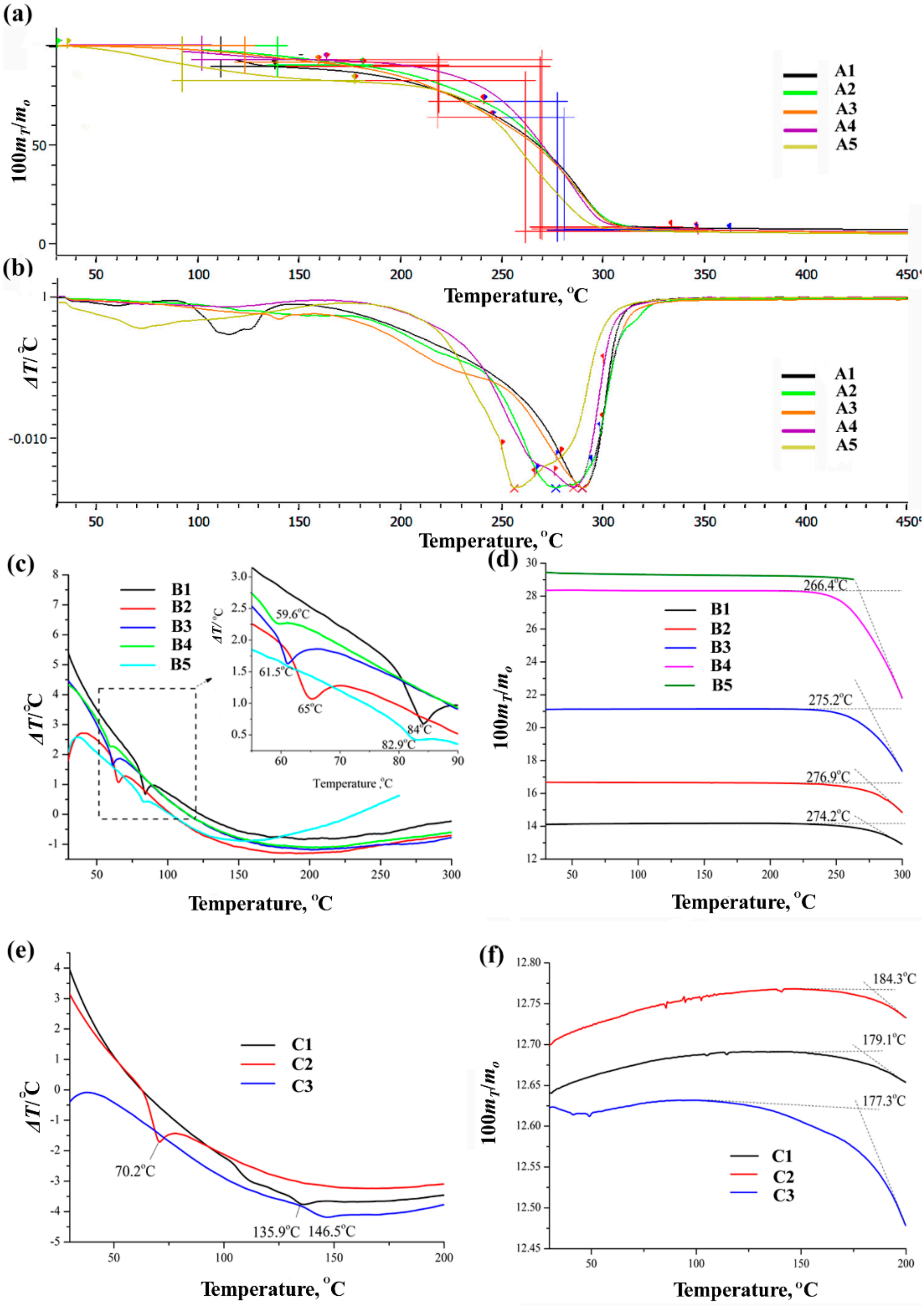

Figure S2. Thermograms of 13 ILs (a: TGA curves of **A1**-**A5**, b: DTA curves of **A1**-**A5**; c: DTA curves of **B1**-**B5**, d: TGA curves of **B1**-**B5**; e: DTA curves of **C1**-**C3**, f: TGA curves of **C1**-**C3**).
